# A proof-of-concept clinical study examining the NRF2 activator sulforaphane against neutrophilic airway inflammation

**DOI:** 10.1186/s12931-016-0406-8

**Published:** 2016-07-22

**Authors:** Charity G. Duran, Allison J. Burbank, Katherine H. Mills, Heather R. Duckworth, Maria M. Aleman, Matthew J. Kesic, David B. Peden, Yinghao Pan, Haibo Zhou, Michelle L. Hernandez

**Affiliations:** UNC Center for Environmental Medicine, Asthma, & Lung Biology, The University of North Carolina at Chapel Hill, Chapel Hill, NC USA; Department of Physical Therapy, Methodist University, Fayetteville, NC USA; Department of Biostatistics, Gillings School of Global Public Health, The University of North Carolina at Chapel Hill, Chapel Hill, NC USA; Division of Allergy, Immunology & Rheumatology, UNC School of Medicine, 104 Mason Farm Road, CB #7310, Chapel Hill, NC 27599-7310 USA

**Keywords:** Sulforaphane, NRF2, Ozone, Airway inflammation, Antioxidants

## Abstract

Sulforaphane (SFN), a naturally occurring isothiocyanate found in cruciferous vegetables, is implicated as a possible therapy for airway inflammation via induction of the transcription factor NF-E2-related factor 2 (NRF2). In this proof-of-concept clinical study, we show that supplementation of SFN with broccoli sprout homogenate in healthy human subjects did not induce expression of antioxidant genes or protect against neutrophilic airway inflammation in an ozone-exposure model. Therefore, dietary sulforaphane supplementation is not a promising candidate for larger scale clinical trials targeting airway inflammation.

**Trial registration:**NCT01625130. Registered 19 June, 2012.

## Introduction

Dear Editor,

Asthma is a heterogeneous chronic disease that can be stratified based on features such as eosinophil or neutrophil predominance, and responsiveness to corticosteroids. Current available therapies including corticosteroids are not as effective for certain forms of the disease, particularly neutrophil-predominant asthma. Furthermore, due to negative perceptions of corticosteroids, the use of complementary and alternative medicine and nutritional interventions for asthma is increasing in the U.S [1]. The naturally occurring isothiocyanate, sulforaphane (SFN), is found in cruciferous vegetables and has been implicated as a possible therapy for airway inflammation via induction of the transcription factor NF-E2-related factor 2 (NRF2), which regulates expression of cytoprotective phase II antioxidant enzymes. The relevance of targeting antioxidant gene expression extends to other airway diseases as well, such as COPD, which is characterized by oxidative stress and dysregulation of antioxidant gene expression [2]. However, there are conflicting reports concerning the ability of SFN to induce antioxidant gene expression, and its effectiveness against airway inflammation [3–5]. In this brief communication, we report our findings from a proof-of-concept study examining if in vivo supplementation with SFN with broccoli sprout homogenate (BSH) is an effective intervention for ozone (O_3_)-induced airway inflammation, a model of neutrophilic airway inflammation. Oxidative injury is especially relevant for those with asthma, as antioxidant reserve may be impaired in this population. O_3_ inhalation causes significant airway neutrophilia in healthy non-asthmatic persons [6], making this a useful model for neutrophilic airway disease.

## Methods

For this randomized, placebo-controlled, crossover study we recruited 16 non-atopic, non-smoking healthy volunteers between the ages of 18–50 years. The O_3_ study protocol was approved by the University of North Carolina’s Institutional Review Board, and written informed consent was obtained from all study subjects. All subjects underwent a standardized screening protocol including allergy skin testing and methacholine challenge as previously described [7, 8]. Volunteers were randomized in a 1:1 ratio to consume either 200 g of BSH (equivalent to 111 g of commercially available Broccosprouts® (Brassica Protection Products LLC)), or 200 g of alfalfa sprout homogenate (ASH), which lacks SFN. The dose of BSH and ASH were chosen based on results of a prior study that found maximal induction of NRF2-dependent gene expression by BSH with a 3 day 200-g dosing regimen [4]. Subjects received supplements once daily for 3 days during the initial study period, and the alternate treatment during the crossover period. On the third day of supplementation, each subject was exposed to O_3_ (0.4 ppm) for 2 h while performing four 15 min sessions of intermittent moderate exercise (defined as minute ventilation or VE_min_ = 30–40 L/min) on a treadmill, separated by 15 min of seated rest. Induced sputum was obtained at screening and at 4 h post-O_3_ exposure and processed for measurement of cytokines and cell counts as previously described [7, 8]. Blood was collected at screening and post-O_3_ for determination of SFN and SFN-conjugate levels by mass spectroscopy. Additionally, blood and nasal epithelial cells (NECs) were collected 4 h post-O3 to measure NRF2-regulated gene expression (HO-1, NQO-1, GSTM-1). There was a minimum washout period of 14 days between treatment periods.

## Results

Our primary hypothesis was that NRF2 activation with SFN would decrease %PMNs in induced sputum compared to placebo after O_3_ exposure. The primary endpoint for this study was the effect of SFN compared to placebo on the O_3_-induced change (post-O_3_ minus pre-O_3_) in %PMNs in airway sputum. To analyze the treatment effect on sputum cellularity, we compared active (BSH) to placebo (ASH) treatment using a linear mixed model approach [9]. Comparisons between post- O_3_ active or placebo treatment to baseline values were carried out using Wilcoxon-Signed rank tests. Criterion for significance was taken to be *p* ≤ 0.05.

**Fig. 1:**
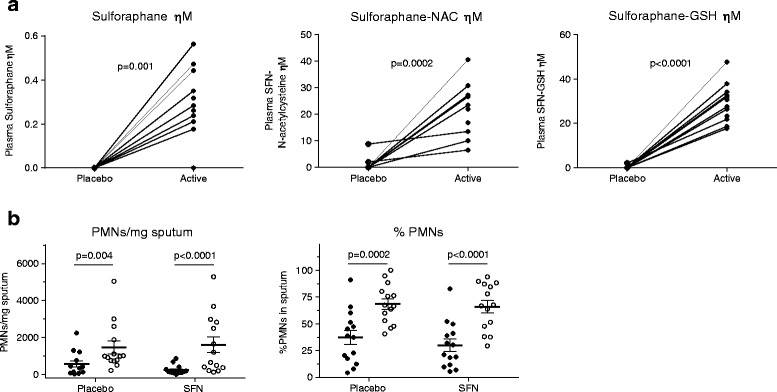
Plasma levels of SFN and its major metabolites SFN-N-acetylcysteine and SFN-glutathione following BSH supplementation (**a**). O_3_-induced changes in sputum neutrophil counts with placebo or SFN supplementation (*N* = 14) (**b**). Changes are presented as PMNs/mg sputum and %PMNs/mg sputum

Sixteen subjects were randomized, and fifteen subjects completed all visits. There were no serious adverse events during the course of the study. Three days of supplementation with BSH significantly increased levels of SFN (*p* = 0.001) and its major metabolites, SFN-*N*-acetyl-L-cysteine (*p* = 0.002) and SFN-glutathione (*p* < 0.001) compared to placebo (Fig. 1a). O_3_ exposure significantly increased the quantity of neutrophils in sputum (expressed as neutrophils/mg and %PMN) in both the placebo and BSH treatment groups (Fig. 1b), but the BSH supplementation group showed no significant difference in sputum neutrophilia compared to placebo. Despite significantly increased plasma levels of SFN in the BSH group, post-O_3_ gene expression of NRF2 and phase II antioxidant defense genes in NECs and peripheral blood were not significantly different from placebo (Table 1).

**Table 1:** % Change antioxidant gene expression in healthy volunteers following O3 exposure

	Placebo	SFN	*p value
Nasal Epithelial Cells			
GSTM1	58.74 ± 57.07	49.99 ± 41.76	0.9375
HO-1	2.041 ± 24.97	−6.06 ± 16.69	0.9097
NQO-1	27.65 ± 44.88	39.35 ± 41.57	0.9097
NRF-2	2.521 ± 17.42	0.9522 ± 18.52	0.9097
Peripheral Blood			
GSTM1	26.29 ± 44.12	−14.11 ± 20.05	>0.9999
HO-1	−5.42 ± 13.93	−7.61 ± 17.17	0.9453
NQO-1	13.87 ± 43.43	−18.18 ± 26.59	0.9375
NRF-2	22.8 ± 27.20	−12.59 ± 10.89	0.6406

Data are shown as mean ± SEM

Changes in GSTM1 expression were performed only on GSTM1 sufficient subjects. For all genes, *N* = 6–14

*Comparisons between Placebo and SFN groups were carried out using paired Wilcoxon-Signed rank tests

## Discussion

SFN has received significant attention in recent years as a possible intervention for oxidant-induced airway inflammation through induction of NRF2-regulated antioxidant genes, but reports concerning its ability to induce antioxidant gene expression and protect against airway inflammation are conflicting. Possible explanations for these contradictory results include variable dosing, dosage forms, and differential biological responses in diseased versus healthy populations. Our study utilized a similar BSH preparation and dosing schedule as Reidl et al., in which 200 g of BSH was ingested daily for three days by healthy volunteers [4]. This preparation reportedly delivered 102 μmol SFN per dose. In contrast to Reidl et al., we saw no differences in phase II enzyme expression in NECs or peripheral blood. Furthermore, BSH supplementation had no impact on lower airway inflammation, as determined by O_3_–induced changes in sputum neutrophilia. Our results are in agreement with Sudini et al., in which ingestion of 100 g of whole broccoli sprouts daily by allergic asthmatics for 3 days had no effect on either NRF2-dependent gene expression in NECs and PBMCs, or eosinophilic lower airway inflammation (measured by FENO) [3]. On the other hand, supplementation of SFN using a standardized dose of broccoli sprout extract inhibited nasal inflammatory responses to diesel exhaust in cat-allergic subjects [5]. These contradictory results may be due to differing systemic levels of SFN achieved with dietary supplementation with BSH, which is not standardized. However, because variability exists in the timing and methods of detection for SFN conjugate levels, it is difficult to compare systemic SFN levels across studies.

Similar to our study, other groups have demonstrated marked increases in SFN conjugate levels following in vivo supplementation with BSH with minimal effects on antioxidant gene expression [3, 10]. It is possible that the plasma levels of SFN achieved with our BSH supplementation regimen were not sufficiently high to be biologically active. A dosing study using fresh broccoli sprouts that achieved significantly higher peak plasma levels of SFN metabolites found no significant increases in antioxidant gene expression in whole blood [10]. Although several in vitro studies report induction of NRF2 genes with SFN treatment, it is important to note that many of these studies utilize concentrations of SFN in the micromolar range [11–14]. Plasma levels of SFN achieved in our study are several orders of magnitude lower than those used in vitro; furthermore, levels achieved in target tissues are likely less than those achieved in plasma. Therefore, doses of BSH that can be reasonably consumed by an adult may exhibit little biologic activity.

## Conclusions

In summary, dietary supplementation of SFN with BSH did not induce expression of NRF2-regulated genes, or have protective effects with O_3_ exposure, a model of neutrophilic airway inflammation. Collectively, these findings suggest that SFN supplementation with BSH is not a promising candidate for larger scale clinical trials targeting airway inflammation.

## Abbreviations

ASH, alfalfa sprout homogenate; BSH, broccoli sprout homogenate; NECs, nasal epithelial cells; NRF2, NF-E2-related factor 2; SFN, sulforaphane
